# Characterization of the complete chloroplast genome sequence of *Actinidia kolomikta*

**DOI:** 10.1080/23802359.2021.1902408

**Published:** 2021-03-19

**Authors:** Dongping Qiu, Ping Tang, Xiaohong Yao

**Affiliations:** a College of Forestry Science and Technology, Lishui Vocational and Technical College, Lishui, China; b Key Laboratory of Plant Germplasm Enhancement and Speciality Agriculture, Wuhan Botanical Garden, The Chinese Academy of Sciences, Wuhan, China

**Keywords:** *Actinidia kolomikta*, chloroplast genome, Actinidiaceae, phylogenetic analysis

## Abstract

The whole complete chloroplast (cp) genome sequence of *Actinidia kolomikta* (formerly *A. maloide*) was sequenced and assembled from Illumina paired-end sequencing. The cp genome of *A. arguta* was 157,425 bp long, containing a large single copy region (LSC) of 88,498 bp and a small single copy region (SSC) of 20,475 bp and a pair of inverted repeat regions (IR) of 242,266 bp. It contained 113 different genes, including 79 protein-coding genes, 30 tRNA genes, 4 ribosomal RNA genes. Phylogenetic analysis using concatenated alignments of whole cp genome sequences revealed that *A. kolomikta* was the sister group to all other groups of *Actinidia*

The genus *Actinidia* Lindl. (Actinidiaceae) comprises 54 species and 21 varieties according to the recent taxonomic revision of Li et al. (2007). However, the taxonomy treatment of *Actinidia* has been controversy and is still under debate due to interspecific hybridization and reticulate evolution in *Actinidia* (Li et al. 2007). The *A. kolomikta* complex contained two species or one variety, *A. kolomikta*, *A. maloide*, *A. maloides* f. *cordata* and *A. tetramera*. Despite the controversy, *A. maloide* and *A. maloides* f. *cordata* mainly distributed in Sichuan and Yunnan Province have been reduced to a synonym of *A. kolomikta* (Deng and Ming 2003). *Actinidia kolomikta* is one of the most widespread species, being found in cooler, northern temperate regions in eastern Russia, Korea, and Japan and throughout much of China from Heilongjiang and Jilin Province in the north to south of the Yangzi River. *Actinidia kolomikta* has very sweet fruit with a fine flavor and, although the fruit are small (2 to 5 g), the plant is recognized as a valuable species for commercial kiwifruit improvement through inter-specific hybridization. However, little is known about the genetic diversity and demographic history of *A. kolomikta*. Knowledge of population genetics information is essential for scientific utilization of germplasm resources for *A. kolomikta*. Although the cp genome of *A. kolomikta* has been previously reported (Lan et al. 2018), additional cp genome of the same species would be useful for developing molecular markers such cp-SSRs and cp-SNPs for population genetics studies. Here, we report the whole complete chloroplast genome of *A. kolomikta* (formerly *A. maloide*), and revealed its phylogenetic position within the genus of *Actinidia* based on the whole cp genome data.

The fresh leaves of a single individual of *A. kolomikta* (formerly *A. maloide*) were collected from Emei mountain, Leshan, Sichuan Province (29˚34΄1.9″ N, 103˚17΄4.1″ E). The voucher was deposited in Herbarium of National *Actinidia* Germplasm Repository of China under the voucher number Acs10077 (Dawei Li, lidawei@wbgcas.cn). Total genomic DNA was isolated using the DNeasy plant Mini Kit (Quiagen, Carlsbad, CA) and stored in a DNA bank of the Wuhan Botanical Garden, the Chinese Academy of Sciences (WBGCAS). The purified DNA (5 μg) was sheared by nebulization with compressed nitrogen, and a short-insert (500 bp) library following the manufacturer’s protocol (Illumina HiSeq 2000) was constructed. After removing adaptor sequences, Illumina paired-end sequencing generated a total of 2,268,024,800-bp raw reads. A total of 44,258,272 (17.2%) out of 251,171,458 reads were mapped to the reference cp genome of *A. chinensis*, generating read coverage depths of 28,307×. The raw reads were assembled via NOVOPlasty (Dierckxsens et al. 2017). The assembled genome was annotated using Geneiousv11.0.3 (Kearse et al. 2012), with the cp genome of *A. chinensis* (Yao et al. 2015) as the reference.

The complete cp genome size of *A. kolomikta* is 157,425 bp, including a pair of IRs (242,266 bp) separated by the LSC of 88,498 bp and SSC of 20,475 bp. The structure of *A. kolomikta* cp genome is very similar to that of other reported *Actinidia* cp genomes. The *A. kolomikta* cp genome contains 113 unique genes, including 79 protein-coding genes, 30 transfer RNA (tRNA) genes and four rRNA genes. There are five protein-coding, eight tRNA and all four rRNA genes duplicated in the IR, and one tRNA gene duplicated in the LSC, making a total of 131 genes present in the chloroplast genomes. Fourteen of the protein-coding genes and eight of the tRNA genes contain introns, almost all of which contain a single intron except for *ycf3*, which has two introns. The average AT content of *A. kolomikta* cp genomes is 62.8%, which is similar to the other published *Actinidia* cp genomes (Yao et al. 2015; Lan et al. 2018; Tang et al. 2019). The cp genome of *A. kolomikta* reported here differs from that of Lan et al (2018) by at least 1,402 chloroplast substitution, 2,599 indels and sequence length (157,425 bp vs 156,875bp).

To reveal the phylogenetic position of *A. kolomikta* in the genus of *Actinidia*, fifteen complete chloroplast genome sequences of Actinidiaceae previously published in the GenBank database were aligned using MAFFT v.7 (Katoh and Standley 2013).

Maximum likelihood (ML) analysis was performed using MEGA7.0 (Kumar et al. 2016) with 1000 bootstrap replicates (Swofford 2003). Phylogenetic relationships with bootstrap values (>50%) are presented in Figure 1. The ML tree indicated that *A. kolomikta* and *A. kolomikta* (formerly *A. maloide*) were closely related and they were the sister group to all other groups of *Actinidia.* The complete plastome reported here can provide valuable information to further reveal the phylogenetic relationships of Actinidiaceae.

**Figure 1.**
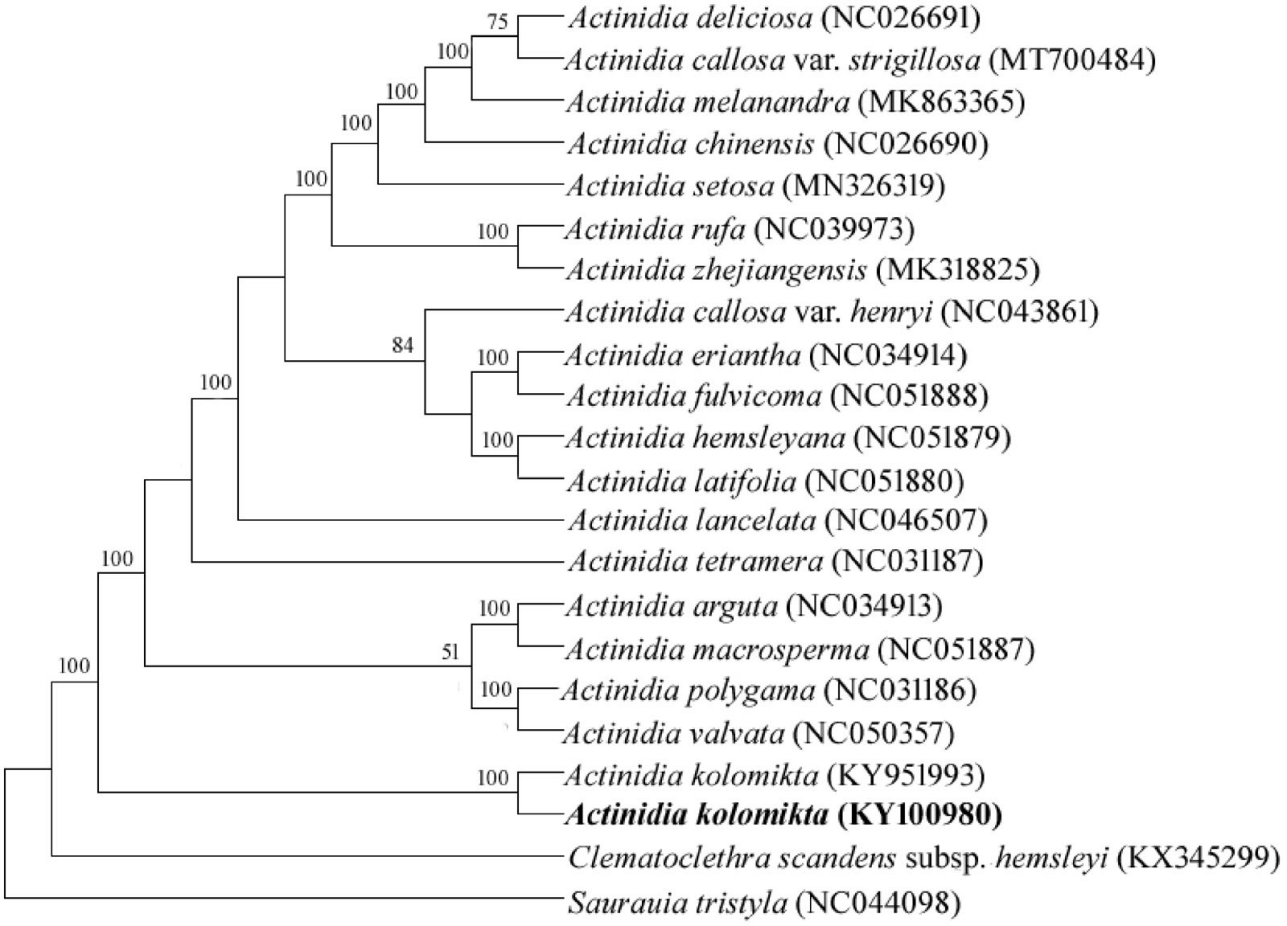
Phylogenetic relathiships of *Actinidia* as inferred by ML analyses of chloroplast genome sequences. Bootstrap value > 50% are given for each clade.

## Data Availability

The genome sequence data that support the findings of this study are openly available in GenBank of NCBI at (https://www.ncbi.nlm.nih.gov/) under the accession no. KY100980. The associated BioProject, SRA, and Bio-Sample numbers are PRJNA318567, SRX1741572, and SAMN04858281, respectively.
